# Health Warnings on Alcoholic Beverages: Perceptions of the Health Risks and Intentions towards Alcohol Consumption

**DOI:** 10.1371/journal.pone.0153027

**Published:** 2016-04-22

**Authors:** Sophie Wigg, Lorenzo D. Stafford

**Affiliations:** Department of Psychology, University of Portsmouth, Portsmouth, United Kingdom; University of Montreal, CANADA

## Abstract

**Objectives:**

Research has demonstrated that packaging which includes pictorial health warnings are more effective in altering smokers’ perceptions and intentions as well as changing smoking behaviours compared to text-only health warnings. However, very few studies have investigated the effectiveness of health warnings on alcoholic beverages

**Methods:**

Participants (N = 60) viewed alcoholic beverages presenting one of three health warnings (No health warning, Text-only, Pictorial) and then responded to questions relating to level of fear arousal and their perceptions toward alcohol use.

**Results:**

We found that pictorial health warnings were associated with significantly higher fear arousal, increased perceptions of the health risks of consuming alcohol as well as greater intentions to reduce and quit alcohol consumption compared to the control.

**Conclusions:**

These novel findings suggest pictorial health warnings on alcoholic beverages may be an important way of making the public aware of the health risks of alcohol consumption.

## Introduction

Alcohol consumption is responsible for 3.3 million deaths worldwide per year and is a causal factor in developing over 200 diseases and injury related conditions [1] including liver cirrhosis, cancers and cardiovascular diseases [2]. In the United Kingdom, alcohol is one of three leading risk factors contributing to disease and death [3], with alcohol related harm estimated to cost the UK over £20 billion annually [4]. Despite research and the national government’s attempts to reduce alcohol consumption, these statistics emphasise the seeming intransigence of the alcohol problem. Thus, new measures to reduce alcohol consumption are urgently needed [5].

One such measure being recommended, is for the introduction of health warnings on alcoholic beverages [6, 7]. In a number of countries health related information on alcoholic beverages is voluntary and may include details on the beverages’ unit content and safe drinking guidelines [8]. The proposed new health warnings on alcoholic beverages would differ significantly and would be similar to those displayed on tobacco packaging. This premise proposes that health warnings displayed on alcoholic beverages would help educate the public about the risks of drinking alcohol [6] and thus aim to change and prevent harmful drinking behaviours [9].

Presently, most countries carry health warnings on tobacco packaging, with the warnings and their contents varying between countries [8, 10]. The effectiveness of such warnings has been explored by investigating perceptions of the health risks of smoking, tobacco related intentions plus behavioural outcomes. Originally, many countries implemented text-only health warnings on tobacco packaging which were shown to be effective in changing smoking attitudes, knowledge and behaviours. For example, research reported that text-only health warnings increased awareness of the health risks of smoking [11]. Additionally, the majority of those who expressed an interest in quitting smoking, agreed that the text warnings on the tobacco packaging had contributed to their decision.

Health warnings on tobacco packaging, combining written warnings and graphic images, known as “pictorial warnings” have also been shown to be effective in altering smokers’ perceptions and tobacco related intentions and behaviours. For instance, one study found a strong positive relationship between thinking about pictorial health warnings and smokers quitting intentions [12]. Also, three months later, those who had read, thought about and discussed the pictorial health warnings were more likely to have quit, attempted to quit or reduced their smoking. Similar findings were also reported in work that compared text-only and pictorial health warnings and found that pictorial warnings were associated with significantly higher quitting motivation [13]. Furthermore, a longitudinal study investigated the effectiveness of pictorial health warnings compared to text-only health warnings on tobacco packaging in Thailand and Malaysia [14]. They reported a reduction in smoking following pictorial health warning exposure compared to control participants. Thus, the authors proposed that pictorial health warnings on tobacco packaging are more effective than text-only health warnings.

The theoretical explanations for these effects include Framing (Prospect Theory) [15, 16], where for instance, loss (compared to gain) framed pictorial warnings on tobacco packaging have been shown to be more effective in reducing intentions to smoke [17]. Research also proposes that the emotional appeal of a health warning may explain its effects [18], where it has been proposed that effective graphic warnings elicit greater emotional responses [19]. In particular, the fear emotion has long been used in communicating health education [20]. These are known as “fear appeals” and are persuasive messages intending to scare by describing the negative consequences of continuing the risky behaviour [21]. A meta-analysis of more than 100 fear appeal articles, proposed that strong fear appeals are perceived as more severe and are more persuasive compared to weak fear appeals [22]. This explanation has been reported in numerous tobacco packaging studies. For example, one study found that highly graphic warnings increased participants’ quitting smoking intentions through fear arousal [23]. Similarly, research reported significantly higher levels of fear arousal and stronger motivations to quit for tobacco packaging containing pictorial health warnings compared to text-only [13]. Tobacco research has been summarised by proposing that pictorial health warnings inducing higher levels of fear are significantly more effective [18].

In comparison to tobacco research, few studies have investigated the effectiveness of health warnings on alcoholic beverages. Over twenty-five years ago, a review reported that the introduction of warning labels in America in 1989 which displayed basic information [24], similar to UK alcoholic beverages, lead to increased awareness of their messages [25]. However, in the 21^st^ century, little research has explored the effectiveness of the proposed new health warnings for alcoholic beverages. Recently, some research has started to examine this issue. For example, Pettigrew and colleagues [26] assessed alcohol consumers’ perceptions towards cancer warning statements on alcoholic beverages. They found health warnings on alcoholic beverages were a potential way of increasing awareness about the causality of alcohol consumption and cancer. It has also been reported that health warnings on alcoholic beverages were shown to increase awareness of alcohol warning label law as well as increasing warning message recall [27]. Glock and Krolak-Schwerdt [28] found health warnings on alcoholic beverages may specifically influence college aged people. Whilst, Al-Hamdani and Smith [7] reported that health warnings on alcohol can change perceptions of alcoholic beverages and highlight the importance of research on alcohol health warnings.

Due to the lack of research investigating the proposed implementation of health warnings on alcoholic beverages, the present study’s aim was to test the effectiveness of a range of alcohol health warnings. Participants were randomly allocated to one of three conditions which varied in the health warning presented on the alcoholic beverage: (1) no health warning [control], (2) text-only, (3) pictorial. The effectiveness of the health warnings were assessed by measuring participants’ perceptions of the health risks of consuming alcohol, their fear arousal and their intentions to reduce and quit alcohol consumption. Based on the tobacco literature [13, 14], it is predicted that the alcoholic beverages displaying the pictorial health warning would significantly increase perceptions of the health risks of consuming alcohol, along with increasing intentions to reduce and quit alcohol consumption compared to the text-only and control conditions. Additionally, based on the “fear appeal theory” and empirical findings [13, 19, 21, 22], it is expected that the pictorial health warnings would significantly increase fear arousal compared to the text-only and control conditions.

## Methods

### Participants

Sixty university students (43 females) participated in the study, aged between 18 and 35 (M = 19.43, SD = 3.10). The participants did not differ in age, gender or their weekly alcohol consumption between the conditions (Table 1). The AUQ revealed that participants total weekly alcohol units consumption (M = 26.97, SD = 14.62) was above the UK recommended weekly intake for both females and males (14 units) [29]. Following this, participants average alcohol consumption levels could be defined as ‘Hazardous’ [29]. Participants were recruited using an online system and were invited to take part only if they were consumers of alcohol and therefore not teetotal. On completion, participants received credit points for participation. The study was approved by the Psychology Ethics Committee at the University.

**Table 1:** Mean (SEM) Participant Characteristics.

	Group						
	Control		Text Only		Pictorial		Group differences
	M	SE	M	SE	M	SE	
Age (N = 60)	19.15	0.70	19.50	0.70	19.65	0.70	F = 0.13, NS
UK alcohol units (p/week) (N = 57)	22.33	3.23	32.26	3.59	26.84	3.08	F = 2.29, NS
Gender (M:F) (N = 60)	4:16		7:13		6:14		Χ^2^ = 1.15, NS
Number of smokers (N = 60)	3		4		5		

### Design

The study used a between-subjects design where participants were randomly allocated to one of three experimental conditions based on the health warning label presented on the alcoholic beverages: No health warning (Control), Text-only, Pictorial. The main dependent variables were the effectiveness of the health warnings including participant’s levels of fear arousal, their perceptions of the health risks of consuming alcohol and their intentions to reduce and quit alcohol consumption.

### Materials

#### Alcohol Usage Questionnaire (AUQ)

Patterns of habitual alcohol consumption were measured using a questionnaire based on [30]. The questionnaire also included questions measuring the number of years participants had regularly drunk alcohol and their planned intentions to quit alcohol consumption.

#### Health warnings and alcoholic beverages

In order to find the most effective pictorial health warning, a preliminary study was completed, where participants (6 females/6 males) rated their fear arousal for three counterbalanced pictorial health warnings which were similar in style to tobacco packaging health warnings and were created by the authors (see appx/online resource). The pictorial images were all sourced from the internet with the rationale for selection based on their connection to alcohol related harm and similar research. Responses were given using a 7-point Likert scale (1 = Not fearful at all, 7 = Extremely fearful), similar in content and style to tobacco packaging health warning research [24, 31]**.** Results demonstrated the highest ratings (M = 5.33, SD = 1.20) for the warning depicting a diseased liver with the text “Alcohol causes fatal liver cancer” which was selected for the main study. In order to maximise authenticity, in the main study, three real alcoholic wine and beer beverages containing full levels of alcohol were used with their original beverage labels replaced with foreign labels to minimise brand familiarity. Mock health warnings were created for the three alcoholic wine and beer beverages which varied in their health warning. One beer and one wine beverage featured no health warning (control condition) and were therefore similar to alcoholic beverages currently available throughout the UK. The second beer and wine beverages featured the text health warning on the front of the beverages (text-only condition) whilst the final beer and wine beverages included the text health warning plus an image of a diseased liver on the front of the alcoholic beverages (pictorial condition). Both the text-only and the pictorial health warnings were of the same size. All package information other than the type of health warning displayed on the beverages (e.g. brand information and the information on the rear of the beverages) was the same across conditions. All labels were created using Microsoft Windows 8 Paint. Labels were in colour, of the same quality and appearance and were printed on plain paper using a HP Deskjet 3000 inkjet printer.

#### Effectiveness of the health warnings on the alcoholic beverages as measured by the product design of alcoholic beverages questionnaire

Due to the absence of previous research, we conducted a pilot study with the main aim being to test the use of the cover story, which was utilized to lead participants away from the true aims of the study. As part of this, the study was described as investigating the product design of alcoholic beverages. Participants (5 females/5 males) completed the AUQ and were then presented with a wine beverage displaying the selected pictorial health warning from the initial pilot. Participants then completed a questionnaire comprising product design items (not analysed) and the main effectiveness measures adopted from tobacco packaging health warning research [13, 14, 19, 23, 32]. An example of the former “How professional do you find the design of the alcohol beverage?”. Results demonstrated the beverages and labels were rated as highly authentic. Using 7-point Likert scales, the main effectiveness measures comprised participant’s indicating their level of fear arousal for the health warning on the alcoholic beverage plus the extent to which the health warning made them think about the health risks of consuming alcohol, their intentions to reduce alcohol consumption and their intentions to quit alcohol consumption as a result of viewing the health warning.

### Procedure

The experiment was conducted in the University’s Department of Psychology. Upon arrival, participants gave informed consent followed by completing the AUQ. Participants were then given the product design of alcoholic beverages questionnaire used in the pilot study along with either a beer or wine beverage which displayed one of three health warnings. On completion, the researcher removed the questionnaire along with the alcoholic beverage. Participants were then presented with a question measuring their warning message recall to check for engagement with reading the label. For this task, they were asked to think about the alcoholic beverage they were presented with and to transcribe what the warning stated. Once finished, the question was removed and participants were given a second alcoholic beverage (beer or wine) which was different to their first presentation. All participants therefore viewed and rated a wine and a beer beverage in the same experimental condition. The presentation of alcoholic beverages (beer and wine) was counterbalanced to minimise order effects. Participants were then given another product design of alcoholic beverages questionnaire. On completion, the alcoholic beverage was removed and participants were given another warning message recall question. After completion, participants were provided with a partial debriefing on the purpose of the study and advised a full debriefing would be communicated to them after completion of the study. A partial debriefing was felt necessary since a full debriefing would have compromised the study for future participants.

### Data Analyses

In order to analyse the warning message recall data, two independent coders who did not know the aim of the experiment were briefed by the researcher. Responses were coded as “accurate” if there was recall of an alcohol problem related to the liver or “inaccurate” if comments referred to alcohol-related diseases not associated with the liver or a “don’t know” response. Results demonstrated that the number of participants who accurately recalled the health warnings was extremely high, with only two participants coded as inaccurate. We decided to include their data since exclusion made no difference to the overall significance of each of the findings. All data cleaning and statistical analyses were performed using SPSS version 21. The data were cleaned whereby values two or more standard deviations from the means were removed from statistical analyses. The data were analysed using MANOVA with the between-subjects factor of warning label (Control, Text-only, Pictorial). Subsequent post-hoc tests were completed with Bonferroni adjustments.

## Results

**Fig 1:**
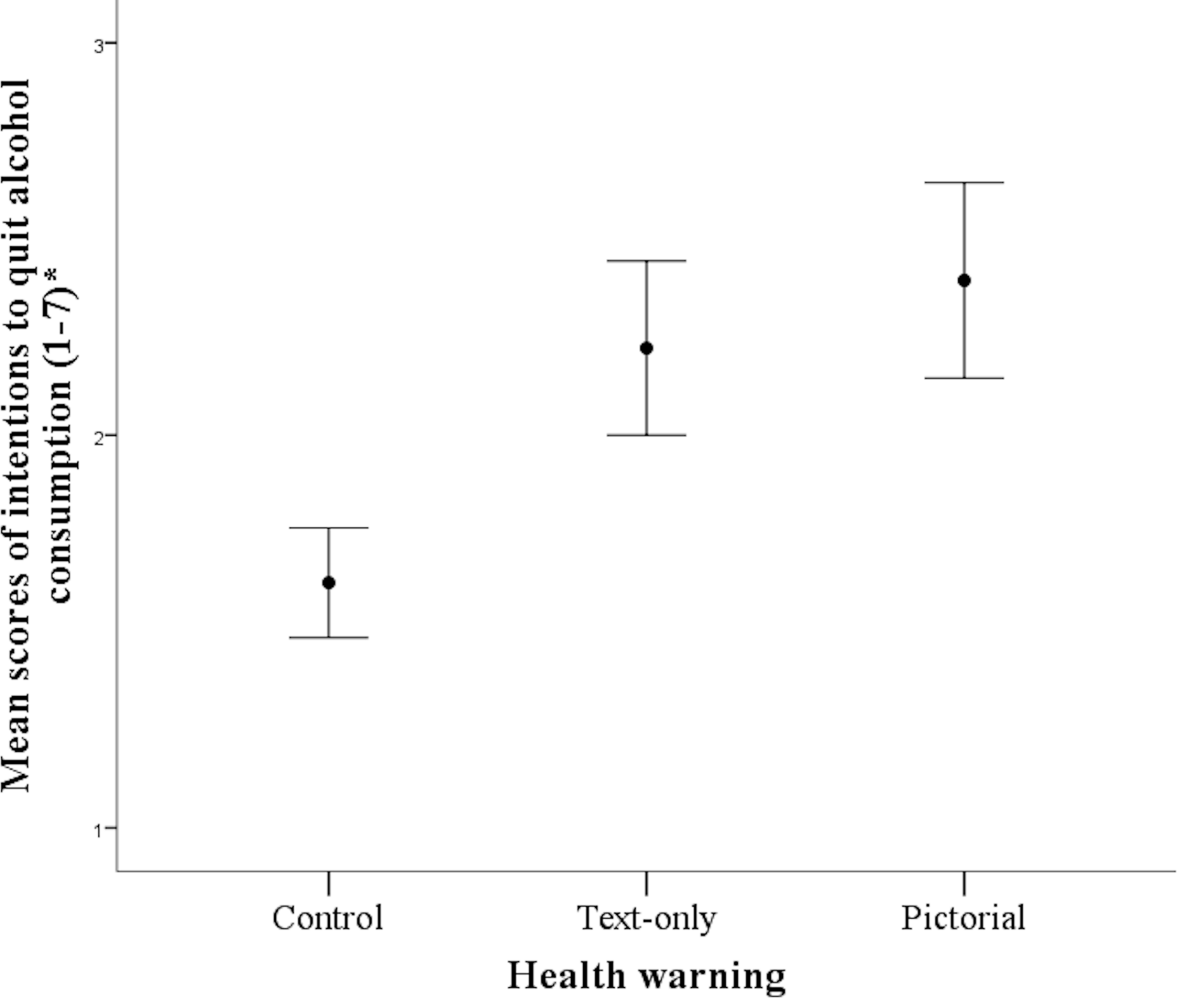
Mean scores of intentions to quit alcohol consumption as a function of health warning. Error bars represent standard errors of the mean.

**Fig 2:**
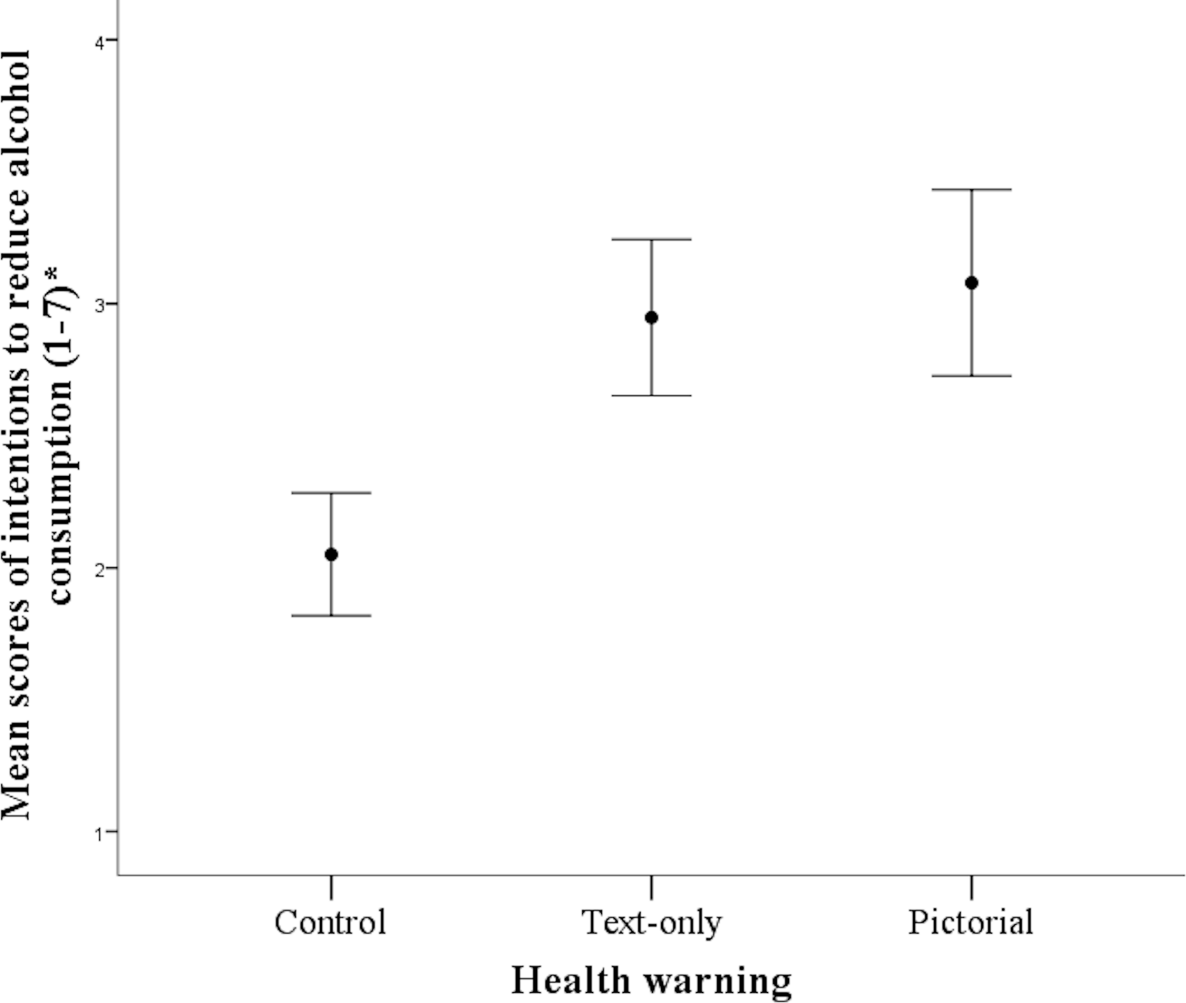
Mean scores of intentions to reduce alcohol consumption as a function of health warning. Error bars represent standard errors of the mean.

Analyses revealed a significant effect of label on all of the measures and consistent with prediction, ratings were highest in the Pictorial condition. For ‘intentions to quit alcohol consumption’, F(2,54) = 3.93, p = .025, ɳ^2^ᵨ = .13 (Fig 1), ratings were higher in the Pictorial versus the Control condition (p = .03). Similarly, for ‘intentions to reduce alcohol consumption’, F(2, 54) = 3.48, p = .038, ɳ^2^ᵨ = .11 (Fig 2), ratings were higher in the Pictorial compared to the Control condition (p = .04). For both of these measurements the comparisons between Pictorial and Text, and Text and Control condition were not significant (all ps >.10). In terms of ‘perceptions of the health risks of consuming alcohol’, F(2, 54) = 6.45, p = .003, ɳ^2^ᵨ = .19, risks were perceived higher in the Pictorial versus the Control (p = .003), and approached significance between the Text and the Control condition (p = .07). The comparison between the Pictorial and the Text conditions was not significant (p = .75). For fear arousal, F(2,54) = 8.97, p < .001, ɳ^2^ᵨ = .25, ratings were higher in the Pictorial (M = 3.84, SD = 1.37) versus the Text (M = 2.89, SD = 1.39) and Control (M = 2.10, SD = 1.08) conditions. Mean comparisons revealed differences between the Pictorial and both the Text (p = .08) and Control (p < .001), with the latter two conditions not differing from each other (p = .19).

### Exposure effects

In order to examine if participants’ perceptions and intentions changed between their first and second exposures to the alcoholic beverages, we completed separate repeated measures ANOVAs for each of the effectiveness measures, using the between-subjects factor of health warning (control/text-only/pictorial) and within-subjects factor of exposure (first/second). Since (from analyses above) we already knew that health warning label had an overall effect on these measures, the main area of interest was to understand if the health warning label x exposure interaction was significant. For fear arousal, results revealed a health warning label x exposure interaction that approached significance, F(1,55) = 3.76, p = .06, ɳ^2^ᵨ = .06, with post hoc analyses demonstrating that fear arousal increased in the control condition (p < .05) but remaining constant for the two remaining conditions (p > 0.4). None of the remaining interactions were significant (Table 2).

**Table 2:** Mean Effectiveness Measures Dependent On Health Warning Condition And Exposure.

Group												
	Control				Text -only				Pictorial			
	Exposure				Exposure				Exposure			
	First		Second		First		Second		First		Second	
	M	SE	M	SE	M	SE	M	SE	M	SE	M	SE
Fear arousal	1.60	0.17	2.60	0.41	3.20	0.39	2.94	0.34	3.70	0.36	3.94	0.31
Perceptions of the health risks of consuming alcohol	2.65	0.40	2.85	0.33	4.40	0.43	3.85	0.45	4.55	0.37	4.55	0.34
Intentions to reduce alcohol consumption	1.80	0.25	2.10	0.31	3.21	0.39	2.68	0.28	3.30	0.40	2.83	0.34
Intentions to quit alcohol consumption	1.35	0.11	1.90	0.24	2.12	0.21	2.17	0.23	2.06	0.25	2.58	0.32

### Correlations

In order to explore the relationship between the main dependent variables and to further our understanding of the possible mechanism behind the effect of health warnings, we completed Pearson correlational analyses. This revealed significant strong relationships for fear arousal to perceptions of the health risks of consuming alcohol and intentions to reduce and quit alcohol consumption (Table 3).

**Table 3:** Pearson Correlation Coefficients Between the Main Effectiveness Measures.

Variable	1	2	3	4
1. Fear arousal	1	.71*	.68*	.66*
2. Perceptions of the health risks of consuming alcohol		1	.58*	.50*
3. Intentions to reduce alcohol consumption			1	.85*
4. Intentions to quit alcohol consumption				1

*p < .01 (2-tailed)

## Discussion

The present study produced several novel findings: participants’ fear arousal, their perceptions of the health risks of consuming alcohol and their intentions to reduce and quit alcohol consumption differed significantly, depending on the health warning label to which they were assigned. Consistent with prediction, the pictorial health warning was found to be the most effective health warning for all of the main measures. Most importantly, we observed that the pictorial health warning led to increased intentions to reduce and quit alcohol consumption compared to the control condition. This finding is in line with related tobacco research [13, 14] which has found pictorial health warnings to be the most effective health warnings in increasing intentions to quit alcohol consumption. Similarly we found that fear arousal and perceptions of the health risks were highest for those participants in the pictorial health warning condition, a finding which is also in keeping with tobacco research [13, 14]. In comparison to tobacco research [13], this study found inconclusive differences between the pictorial and the text-only conditions in the main effectiveness measures, however, since the pictorial health warning yielded the clearest effects compared to control, the results were therefore in the direction expected.

In terms of the relationships between the main effectiveness measures, as demonstrated in tobacco research, perceptions of the health risks were associated with intentions to quit [12]. Also, consistent with tobacco research, associations were found between fear arousal and intentions to quit [13, 23]. The significant positive associations found between fear arousal and perceptions of the health risks and intentions to reduce and quit alcohol consumption lends support to the “fear appeal theory”. Additionally these relationships suggest the role of induced fear as an underlying mechanism for the effectiveness of the health warnings. Given the mediation of fear arousal, stronger fear appeals arousing higher levels of fear help to explain why the text-only and pictorial health warnings were more effective than the control in increasing perceptions of the health risks of consuming alcohol and increasing intentions to reduce and quit alcohol consumption. Following this, the highest levels of fear arousal induced by the pictorial condition provides an explanation for why this was the most effective health warning.

These findings also extend our understanding of applying Prospect theory [15, 16] in the alcohol domain, since it permitted a test of the effects of Framing alone (text-only) and together with a graphical representation (pictorial). Since we found no difference in the text-only versus control condition, this suggests that both a Framing health warning text message plus a pictorial image are needed to generate differences in these key measures. It could further be theorised that, as suggested by tobacco research, the pictorial image acted to increase attention towards the health warning text which in turn increased their effectiveness [18].

In terms of exposure effects, no significant differences were found in the main effectiveness measures between participants’ first and second exposures to the health warnings. This may suggest the effectiveness of pictorial health warnings following repeated exposures to alcoholic beverages. Research examining the effects of repeated exposures to pictorial health warnings on tobacco packaging following their implementation in Canada reported finding very little ‘wear out’ effect more than a year after their introduction [12]. The continued effectiveness was suggested to be due to the use of different health warnings presented on the tobacco packaging. Further tobacco research supports this, suggesting that different health warnings which are rotated increase their effectiveness [33, 34]. More recently, Li and co-workers [35] proposed the importance of the warnings size compared to the type of warning in increasing their effectiveness, although both may work collectively. Findings from the present study and tobacco research therefore suggest that pictorial health warnings presented on alcoholic beverages may sustain their effectiveness following repeated exposures.

Considering the limitations of the study, we acknowledge that the use of an experimental design decreases ecological validity, but contend that this needs to be set against the increased knowledge the current study has delivered. One of the strengths of the study was the use of commercially available alcohol beverages and warning labels rated as highly authentic in the pre-study. We therefore believe this provides a realistic insight into the effects that may be observed if health warnings are mandated on alcoholic beverages. It also needs to be noted that the differences in our study were between pictorial health warnings compared to a control of the health messages currently contained on beverages in the UK. Hence we cannot be certain if the effects would be maintained if we had used a control containing an emotionally arousing (e.g. IAPS, [36]) image, not directly connected to alcohol use. However, it needs to be recalled that the ultimate point of such health warning messages is to help educate the public on the connection between the health consequences of excess use. Hence, even if similar effects were found for an emotionally arousing image unconnected to alcohol, it would not negate the use of the more accurate image connected to alcohol consumption.

Research has reported that alcohol consumption in moderation may be beneficial somatically [37] and thus questions the need for health warnings on alcoholic beverages. Interestingly however, the most recent UK government guidelines, based on the latest scientific evidence state the only benefits of alcohol in terms of heart function, are for a very small minority (females aged over 55yrs consuming <5units per week); whereas there are adverse effects related to cancer at any level of alcohol consumption [29]. In summary, the state of knowledge points towards the benefits of using health warnings on alcoholic beverages.

It is acknowledged that alcohol can be consumed without drinkers seeing the alcohol packaging, for example in bars/pubs and thus questions the effectiveness of health warnings on alcohol packaging. Yet, research has shown that alcohol purchased from supermarkets is more than twice the level of alcohol consumed in bars/pubs [38]. Therefore, the majority of drinkers will view the health warnings. For the proportion of alcohol which is consumed in bars/pubs, perhaps modifications could be made to bar taps whereby a health warning is displayed.

The current study was conducted using a majority of female participants and therefore did not permit a meaningful analyses of gender differences. It is therefore uncertain whether the same effects would be observed for male participants. Interestingly, related research has shown that women perceived cancer warning statements on alcoholic beverages as more believable and convincing compared to males [26], which could suggest that the effects seen here would be weaker in males. We also recognise that the use of health warning labels, though important, are just one of a number of factors in discouraging hazardous consumption that can also include mass media campaigns, tax increases, alcohol-free policies [39] plus education [31] and treatments to help people quit alcohol [40].

In summary, in the first study to assess the effectiveness of mandated health warnings on alcoholic beverages, we found that individuals exposed to pictorial health warnings had greater intentions to reduce and quit alcohol consumption than control conditions. These findings provide support that health warnings can be effective in increasing perceptions of the health risks of consuming alcohol and increasing intentions to reduce and quit alcohol consumption.

## Supporting Information

S1 File AUQ. (PDF)

S2 File Main Questionnaire 1. (PDF)

S3 File Main Questionnaire 2. (PDF)

S4 File Pilot study images. (PDF)

S5 File Bottle labels. (PDF)

S6 File Main Effectiveness measure data. (XLSX)
