# Supplementary material for: Health Warnings on Alcoholic Beverages: Perceptions of the Health Risks and Intentions towards Alcohol Consumption
Source: PLoS One. 2016 Apr 22;11(4):e0153027. doi: 10.1371/journal.pone.0153027 (PMC4841515; doi:10.1371/journal.pone.0153027)
Supplement: S1 File — (PDF) [file pone.0153027.s001.pdf]

Alcohol usage questionnaire (AUQ)

Product design of alcohol beverages

Participant number:

Basic information:

- Age:…………
- Gender:………………………

The following questions ask you about your habitual use of different types of alcoholic beverages. Please consider your drinking for the last six months in answering these questions, and take your time to give an accurate response to each one.

1. On how many days each week do you drink wine or any wine based drink (e.g. port, sherry) ? ………..

   Please detail your usual brands …………………………………………………

2. On those days you do drink wine (or similar), roughly how many glasses do you drink ?

   Small Glass 125ml [______] Standard Glass 175ml [______] Large Glass 250ml [______]

3. If you are not sure, please estimate the number of bottles or parts of a bottle …….

4. On how many days per week do you drink beer or cider (at least half a pint) ? ……..
   Please detail your usual brands …………………………………………………

5. On those days you do drink beer or cider, roughly how many pints do you drink ? ………..

6. On how many days per week do you drink spirits (vodka, whisky, gin etc) ? ……..
   Please detail your usual brands …………………………………………………

7. On those days you do drink spirits, roughly how many shots (standard single pub measures) do you drink ? ………..

8. If you are not sure, please estimate the number of bottles or parts of a bottle ? …….

9. On how many days per week do you drink alcopops? ……………
   Please detail your usual brands (e.g. Smirnoff Ice, Moscow Mule, Bacardi Breezer) …………………………………………………

10. On those days you do drink alcopops, roughly how many bottles do you drink ? ………..
11. When you do drink how fast do you drink. For this question, a drink is equivalent to a glass of wine, a pint of beer, a shot of spirits (straight or mixed) or an alcopop. Please circle one of the options:

7 or more drinks per hour

6 drinks per hour

5 drinks per hour

4 drinks per hour

3 drinks per hour

2 drinks per hour

1 drink per hour

1 drink in two hours

1 drink in three or more hours

12. How many times have you been drunk in the last six months ? By drunk we mean loss of co-ordination, nausea, and/or inability to speak clearly ? …………….

13. What percentage of the times you do drink do you get drunk ? ………………

14. Do you smoke ? Yes [__] No [__]
    If yes, on average how many cigarettes do you smoke per day ? ………………

15. Please state the number of years you have regularly drank alcoholic beverages…………………

16. (Please circle one response) I am planning to quit consuming alcohol in the next:

30 days 3 months 6 months 1 year not at all
