# Supplementary material for: Health Warnings on Alcoholic Beverages: Perceptions of the Health Risks and Intentions towards Alcohol Consumption
Source: PLoS One. 2016 Apr 22;11(4):e0153027. doi: 10.1371/journal.pone.0153027 (PMC4841515; doi:10.1371/journal.pone.0153027)
Supplement: S2 File — (PDF) [file pone.0153027.s002.pdf]

Product design of alcohol beverages questionnaire for text-only and pictorial conditions

Participant number:

You will now be given an alcoholic beverage. Please view the front and rear of the alcoholic beverage and complete the questions/statements below.

For each of the following questions or statements, please circle one response.

1. How pleasing do you find the design of the alcohol beverage?

1	2	3	4	5	6	7
Not at all pleasing						Extremely pleasing

2. How professional do you find the design of the alcohol beverage?

1	2	3	4	5	6	7
Not at all professional						Extremely professional

3. How eye catching are the colours used on the design of the alcohol beverage?

1	2	3	4	5	6	7
Not at all eye catching						Extremely eye catching

4. How important to you is the feel of an alcohol beverage in a glass or bottle/can?

1	2	3	4	5	6	7
Not at all important						Extremely important

5. What is the likelihood of you purchasing this alcohol beverage based on its design?

1	2	3	4	5	6	7
Not at all likely						Extremely likely

6. Please state how much you would pay for this alcohol beverage:

   £………………

7. Below in as much detail as possible please describe the most important aspect or aspects to you of the design of alcoholic beverages:

…………………………………………………………………………………………
…………………………………………………………………………………………
…………………………………………………………………………………………
…………………………………………………………………………………………
…………………………………………………………………………………………
…………………………………………………………………………………………
…………………………………………………………………………………………..

8. Below please suggest any improvements which you think may help to enhance the design of this alcohol beverage:
   …………………………………………………………………………………………
   …………………………………………………………………………………………
   …………………………………………………………………………………………
   …………………………………………………………………………………………
   …………………………………………………………………………………………
   …………………………………………………………………………………………
   …………………………………………………………………………………………
   …………………………………………………………………………

For each of the following statements, please circle one answer which is most relevant to you.

9. The health warning on the alcohol beverage makes me think about the health risks (health danger) of consuming alcohol:

   1 2 3 4 5 6 7

   Strongly disagree Strongly agree

10. The health warning on the alcohol beverage accurately depicts the risk to my health:

    1 2 3 4 5 6 7

    Strongly disagree Strongly agree

11. The health warning on the alcohol beverage makes me feel:

    1 2 3 4 5 6 7

    Not fearful at all Extremely fearful

12. The health warning on the alcohol beverage makes me more likely to reduce my daily alcohol consumption:

    1 2 3 4 5 6 7

Strongly disagree Strongly agree

13. The health warning on the alcohol beverage makes me more likely to quit consuming alcohol:

☐ 1 ☐ 2 ☐ 3 ☐ 4 ☐ 5 ☐ 6 ☐ 7

Strongly disagree Strongly agree

14. The information presented on the alcohol beverage would help me quit consuming alcohol:

☐ 1 ☐ 2 ☐ 3 ☐ 4 ☐ 5 ☐ 6 ☐ 7

Strongly disagree Strongly agree

15. I have confidence in my ability to quit consuming alcohol:

☐ 1 ☐ 2 ☐ 3 ☐ 4 ☐ 5 ☐ 6 ☐ 7

Strongly disagree Strongly agree

Please advise the researcher once you have completed all of the questions/statements.

Participant number:

Please think about the alcohol beverage you were presented with. Below, try to recall what the warning on the alcohol beverage stated:

……………………………………………………………………………………………
……………………………………………………………………………………………
……………………………………………………………………………………………
……………………………………………………………………………………………
……………………………………………………………………………………………
……………………………………………………………………………………………
