# Supplementary material for: Health Warnings on Alcoholic Beverages: Perceptions of the Health Risks and Intentions towards Alcohol Consumption
Source: PLoS One. 2016 Apr 22;11(4):e0153027. doi: 10.1371/journal.pone.0153027 (PMC4841515; doi:10.1371/journal.pone.0153027)

Pilot study pictorial health warnings

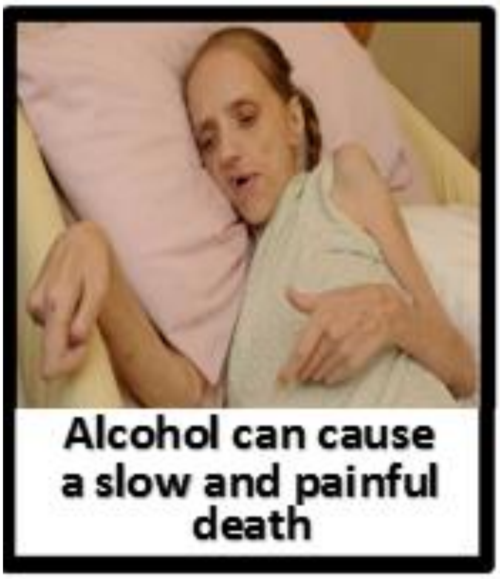

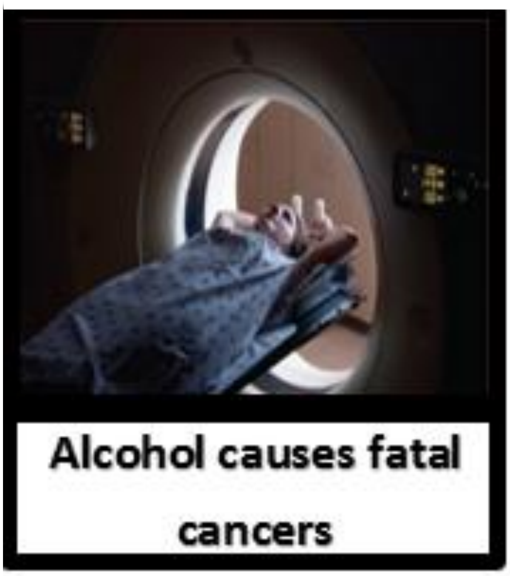

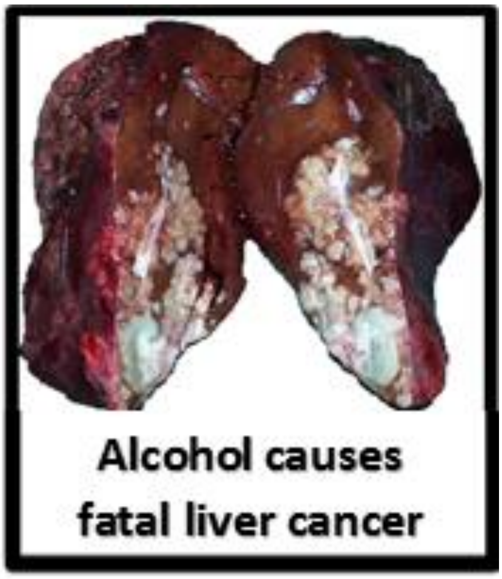

Supplement: S4 File — (PDF) [file pone.0153027.s004.pdf]
