# Supplementary material for: Health Warnings on Alcoholic Beverages: Perceptions of the Health Risks and Intentions towards Alcohol Consumption
Source: PLoS One. 2016 Apr 22;11(4):e0153027. doi: 10.1371/journal.pone.0153027 (PMC4841515; doi:10.1371/journal.pone.0153027)

Front and rear of wine beverages used in the main study (from left to right: pictorial, text-only and control)

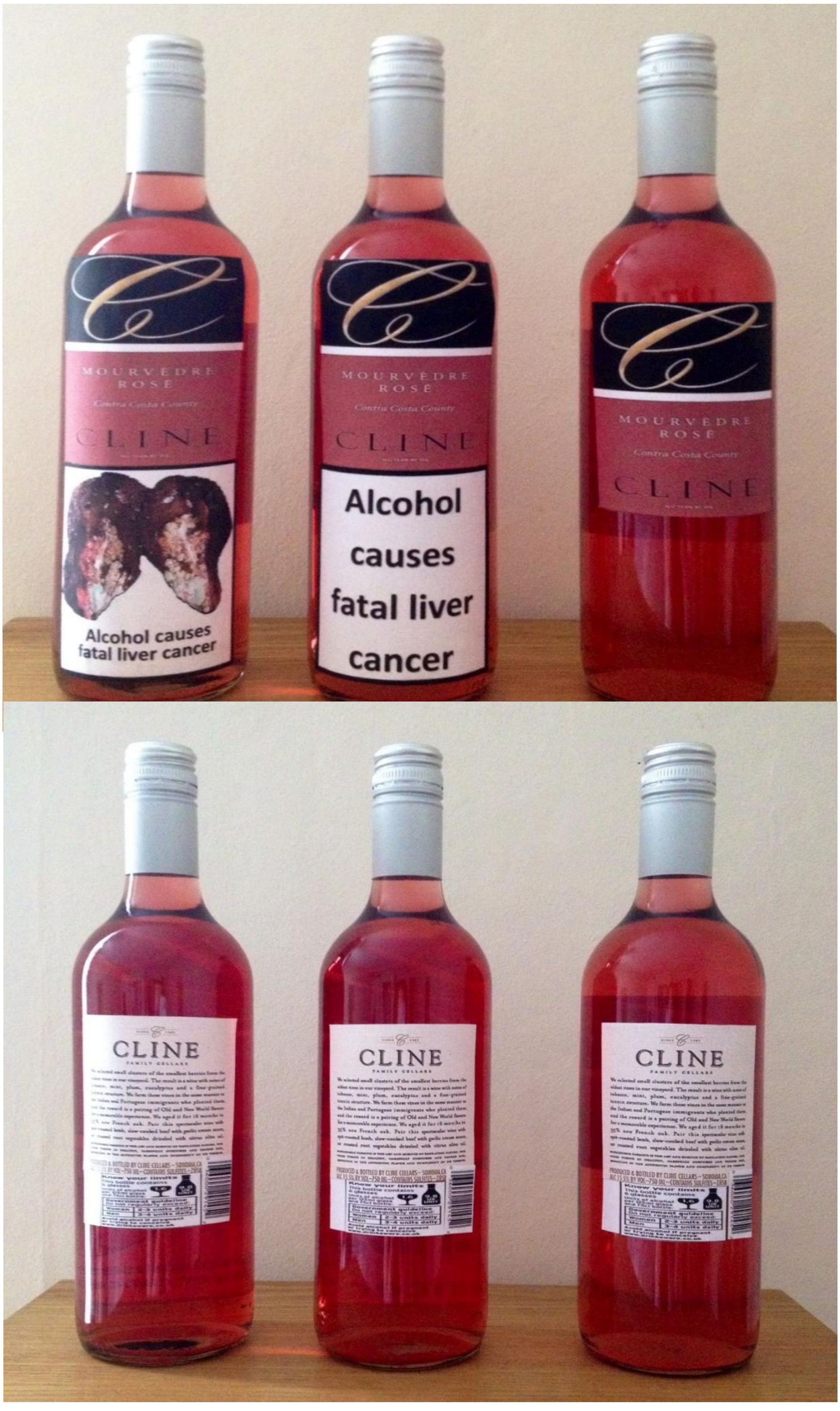

Front and rear of beer beverages used in the main study (from left to right: pictorial, text-only and control)

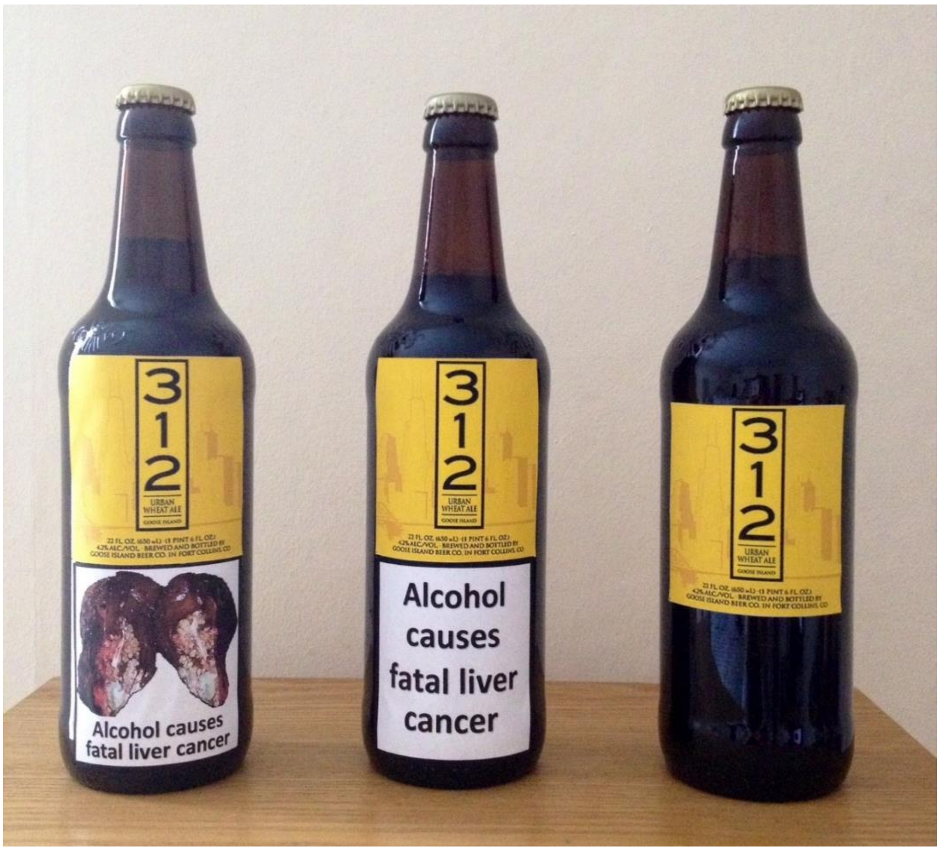

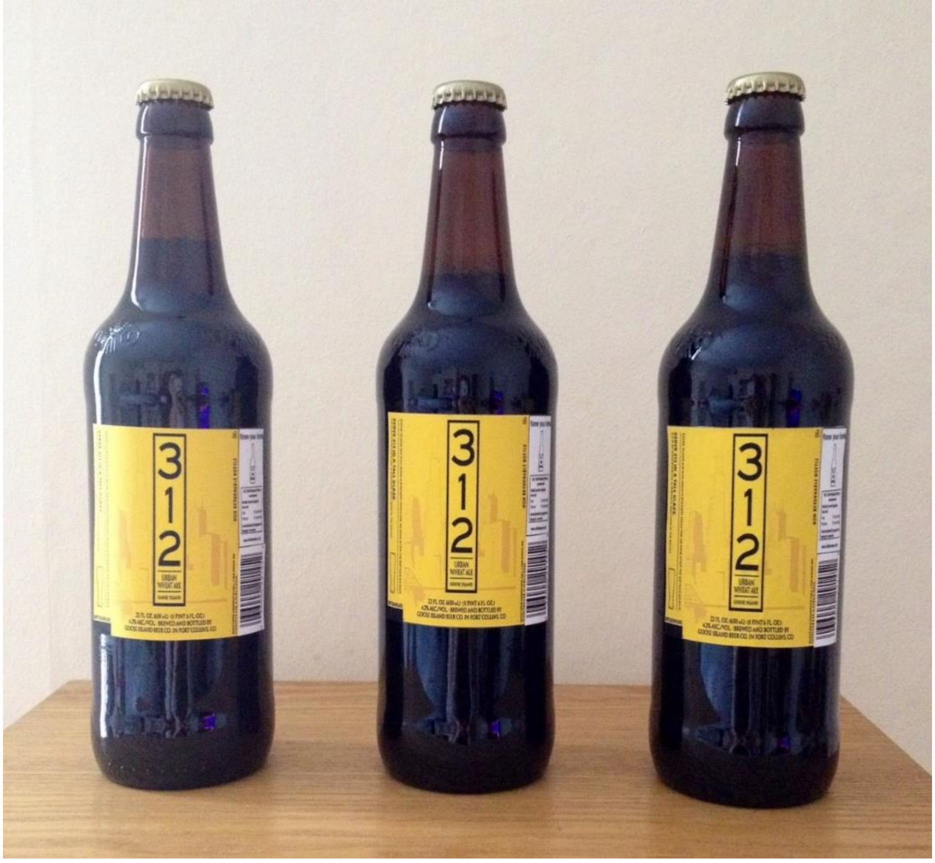

Supplement: S5 File — (PDF) [file pone.0153027.s005.pdf]
